# Temperature Dependence of Thermal Properties of *Ex Vivo* Porcine Heart and Lung in Hyperthermia and Ablative Temperature Ranges

**DOI:** 10.1007/s10439-022-03122-9

**Published:** 2023-01-19

**Authors:** Leonardo Bianchi, Martina Bontempi, Sabrina De Simone, Martina Franceschet, Paola Saccomandi

**Affiliations:** Department of Mechanical Engineering, Politecnico di Milano, 20156 Milan, Italy

**Keywords:** Heart, Lung, Thermal properties, Temperature dependence, Hyperthermia, Thermal ablation

## Abstract

This work proposes the characterization of the temperature dependence of the thermal properties of heart and lung tissues from room temperature up to > 90 °C. The thermal diffusivity (*α*), thermal conductivity (*k*), and volumetric heat capacity (*C*_*v*_) of *ex vivo* porcine hearts and deflated lungs were measured with a dual-needle sensor technique. *α* and *k* associated with heart tissue remained almost constant until ~ 70 and ~ 80 °C, accordingly. Above ~ 80 °C, a more substantial variation in these thermal properties was registered: at 94 °C, *α* and *k* respectively experienced a 2.3- and 1.5- fold increase compared to their nominal values, showing average values of 0.346 mm^2^/s and 0.828 W/(m·K), accordingly. Conversely, *C*_*v*_ was almost constant until 55 °C and decreased afterward (*e.g.*, *C*_*v*_ = 2.42 MJ/(m^3^·K) at 94 °C). Concerning the lung tissue, both its *α* and *k* were characterized by an exponential increase with temperature, showing a marked increment at supraphysiological and ablative temperatures (at 91 °C, *α* and *k* were equal to 2.120 mm^2^/s and 2.721 W/(m·K), respectively, i.e., 13.7- and 13.1-fold higher compared to their baseline values). Regression analysis was performed to attain the best-fit curves interpolating the measured data, thus providing models of the temperature dependence of the investigated properties. These models can be useful for increasing the accuracy of simulation-based preplanning frameworks of interventional thermal procedures, and the realization of tissue-mimicking materials.

## Introduction

The characterization of thermo-physical properties of biological tissues has a key role in several branches of medical, biological, and bioengineering applications.^12^ Among the various physical parameters, thermal properties have gained a huge interest due to their utilization in critical fields in which the quantitative understanding of short- and long-term thermal effects on tissues is pivotal for the optimal design of monitoring and therapeutic devices, and a deeper investigation of the biological phenomena. Indeed, tissue thermal properties are widely employed in simulations of hyperthermal and thermal ablation procedures for cancer treatment,^13,44^ analysis of the heat transfer between implantable devices, ablation catheters and the body,^32,36,38,60^ modeling of the heat transport during physiological mechanisms^30,54^ and to realize tissue-mimicking phantoms.^6^

The effects of the temperature on the tissue depend upon the range of temperature variation and the permanence of the medium at a specific temperature. In general, we can categorize the temperature effects on the biological tissues as follows. When the tissue temperature is increased until 42 °C to 45 °C for around 30 min, the blood flow and vasodilation are usually augmented, thus dispersing heat to protect cells from thermal damage;^52^ afterward, a decrease in blood flow has been observed in several tissues.^12^ In this range, also the metabolism of the cells is modified, indeed, cells require a higher amount of energy to guarantee basic conditions, such as ion gradients across cell membranes, and to preserve structural and physical properties.^42^ For temperatures higher than 45–50 °C, the damage starts to be irreversible.^18^ To obtain long-term benefits, it is required to exceed 60 °C, leading to the denaturation of proteins and instantaneous cell death. For temperatures above 80 °C, physical changes in the tissue occur due to phenomena such as the evaporation of water. All these phenomena are accompanied by quantitative temperature-dependent variations of the macroscopical properties of the tissues.

While with implantable devices it is important that body temperature does not exceed the limit of almost 42 °C to avoid unwanted damage, during thermal therapies the tissue temperature has to increase in the range of 50–100 °C, until the required therapeutic levels are met.^2^ Thermal ablation therapies include high-intensity focused ultrasound, radiofrequency, microwave, and laser ablation, which are the typical thermal ablation strategies used for localized tumor treatment.^53^ They can be performed through minimally invasive approaches, and have shown to be effective in treating malignant cells from a large group of cancers and in various biological tissues such as liver, kidney, and lungs.^17,59^

Thermal therapies for localized malignancies are mainly introduced to treat not operable patients. In the specific case of lung, 70–80% of the patients cannot undergo surgery, and image-guided thermal ablation can represent a good minimally invasive alternative to treat patients with early-stage primary lung cancer, oligometastasis, or local recurrence.^37^ Radiofrequency and microwave ablation are the most used thermal ablation techniques for inoperable lung cancer.

Ablation is also effective in cases of arrhythmias or atrial fibrillation because the cells responsible for the propagation of the dysrhythmia are destroyed.^1,5^ At ablative temperatures, the target tissue undergoes irreversible coagulation necrosis and then evolves into nonconducting myocardial scar tissue, being this therapy definitive compared to drug therapy.^57^

Cardiac ablation is nowadays a routine procedure that provides patients with arrhythmias relief from symptoms and results in an improvement in quality of life.^23^

In both these cases, the aim is to destroy pathological cells and to preserve, at the same time, the surrounding healthy tissue and anatomical structures. The limited experimentation in this field, mostly motivated by ethical reasons, and the poor standardization in the clinical practice make the role of heat transfer modeling and tissue-mimicking phantoms strategic for the analysis of the problems and the optimization of the procedures.

The knowledge of the thermal properties of target tissues and their values as a function of temperature is required for the accurate prediction of the thermal effects in all the mentioned scenarios. Indeed, the amount of delivered heat and damage are strictly related to the temperature distribution in the biological medium. The way the temperature distributes within the tissue is influenced by the tissue's thermal properties, such as thermal conductivity (*k*) [W/(m·K)], thermal diffusivity (*α*) [mm^2^/s], and volumetric heat capacity (*C*_*v*_) [(MJ/(m^3^·K)], which govern the heat transfer, and also vary with the local temperature.^4,6,12^ The *k* describes the energy transfer by conduction in tissue in the steady state. The *α* is instead related to the nonsteady state and refers to the capability of the biological media to conduct thermal energy with regard to reserve heat.^16,46^ *C*_*v*_ is defined as the ratio between *k* and *α*, and it represents the amount of energy (heat) that must be added to cause an increase of one unit in temperature, per unit volume. The relationship among these properties is defined by the following equation:

1 $$ \alpha = k/\left( {\rho c} \right) = k/C_{v} $$

being *ρ* and *c* the tissue density and the specific heat of tissue, respectively.^43,46^

Over the last 40 years, many studies have been conducted to analyze the thermal properties of biological tissue.^25,39,47,49,50^ One of the first and wide investigations was performed by Valvano *et* *al.*, who measured *k* and *α* of different *ex vivo* biological tissues, including heart and lung, in the temperature range between 3 and 45 °C, using a self-heated thermistor inserted into the tissue. The results showed there was considerable variation in the thermal properties from tissue to tissue.^55^ Other studies have employed thermodynamic analysis based on Differential Scanning Calorimetry, to measure only the specific heat capacity of some tissues (like Baker’s cyst) *versus* temperature change.^19^A few other studies have been carried out to estimate the thermal properties of relevant organs, such as heart and lung, and in most cases, the temperature range did not cover higher temperature values (*e.g.*, beyond 76 °C)^10,48^ which are reached during localized thermal therapies on these organs.^8,29,51^ Hence, motivated by the limited data on heart and lung, and by the huge demand for accurate models for describing the heat transfer in these organs, this paper focuses on the measurement of the thermal properties of these tissues, from room temperature up to 94 °C. The thermal conductivity, thermal diffusivity, and volumetric heat capacity of *ex vivo* porcine heart and lung were measured with a dual-needle sensor technique, which has been previously validated by our group on other organs.^43^ The behavior of the measured thermal properties of the heart and lung tissues as a function of temperature has been described by tissue-specific curves, and the analysis of the measurement repeatability has been carried out.

## Materials and Methods

### Experimental Setup

**FIGURE 1:**
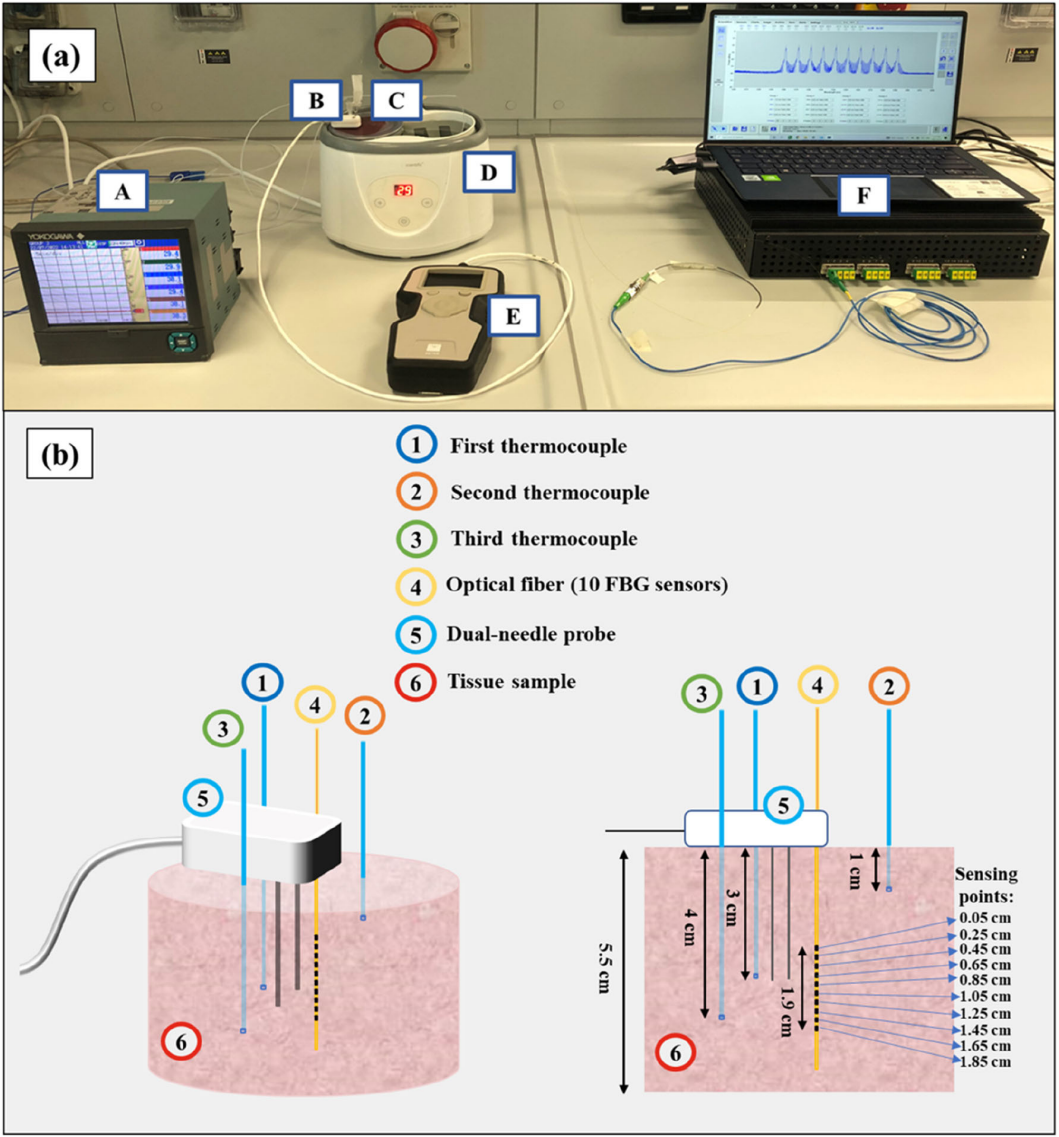
(a) Experimental setup utilized for the measurement of the thermal properties of heart and lung tissues as a function of temperature: (A) data acquisition system used for the monitoring of the temperature registered by the thermocouples, (B) dual-needle SH-3 sensor probe: the sensor is composed of two needles, 30 mm in length, 1.3-mm diameter with 6 mm spacing, for heating the tissue and sensing the subsequent temperature variation; it can operate in a range of temperatures between − 50 and 150 °C, and it is connected to an analyzer of thermal properties to allow for the measure of *k*, *C*_*v*_ and *α* in solid and granular materials, (C) tissue sample, (D) water thermal bath, (E) thermal properties analyzer, (F) fiber optic-based thermometric system including the optical interrogator employed to interrogate the FBG sensors and recover the optical information, thus the tissue temperature variation. (b) Position of the dual-needle probe, thermocouples, and fiber optic sensors within the tissue sample: three-dimensional view (left) and side view (right); the sensing points related to the FBG sensors are also shown.

The experimental setup employed to measure the thermal properties of the deflated lungs and heart tissues consists of (Fig. 1a):

- a water thermal bath (IVYX Scientific Laboratory Digital Water Bath, 20–100 °C temperature range, temperature fluctuation: 0.5 °C, rated wattage: 200 W, possibility of fast ramp-up from 20 to 37 °C in 10 min) used to control the water temperature in the range of interest (from room temperature to > 90 °C);
- a thermally conductive metallic container filled with the sample, suitably sealed to avoid direct contact between the specimen and the water of the thermal bath;
- four thermocouples (associated with a temperature monitoring module, Yokogawa FX1000 Paperless Recorder) for water and sample monitoring;
- TEMPOS thermal properties analyzer with an SH-3 dual-needle sensor probe;
- a metallic needle instrumented with 10 temperature sensors based on Fiber Bragg Grating (FBG) technology.

For both the heart and lung tissue specimens, some precautions were taken to guarantee an adequate volume of tissue to fill the metal container, ensuring, in particular, that the needles of the dual-needle probe were surrounded by at least 15 mm of tissue.^24^ For these reasons, each heart was divided into two portions, discarding not homogeneous parts of the sample like valves and blood clots. The lung samples were prepared by removing the trachea and avoiding bronchi, bronchioles, and alveoli.

Each sample was arranged inside the metal container covered with a silicone lid previously fixed with parafilm to prevent direct contact between the tissue and the water. The container was inserted into the thermal bath to allow the tissue to heat up and reach the desired temperature, maintaining it throughout the experimental procedure. The lid was drilled with six holes: two for the SH-3 dual-needle sensor, one for the 10-temperature-sensors metallic needle based on FBG technology, and three for the thermocouples. The thermal properties were measured using a commercial analyzer (TEMPOS, Meter Group, Inc., Pullman, WA, USA, accuracy: 10%)^27^ with an SH-3 dual-needle sensor. This sensor, which operates in a range of temperatures between − 50 and 150 °C, is connected to the analyzer of thermal properties and can measure *k*, *C*_*v,*_ and *α* in solid and granular materials. The sensor is composed of two needles, the heating needle and the measuring one, both 30 mm in length, placed 6 mm away from each other and with a diameter of 1.3 mm.

The thermal properties of a reference material (i.e., white plastic Delrin® cylinder in polyoxymethylene) were measured to validate the accuracy of the used measurement system and assure that it was functioning in accordance with the specifications. At room temperature (i.e., 22–25 °C), the thermal properties of the standard material provided by the manufacturer are 0.384 W/(m·K), 0.189 mm^2^/s, and 2.03 MJ/(m^3^·K) for *k*, *α*, and *C*_*v*_, respectively; we measured values equal to 0.386 W/(m·K), 0.193 mm^2^/s, and 2.00 MJ/(m^3^·K) for *k*, *α*, and *C*_*v*_, accordingly (maximum percentage difference of 2% compared to the values provided by the manufacturer). Hence, the utilized system was demonstrated to operate properly and in absence of event flaws.

### Thermal Properties Measurement Method

The operating mechanism of the instrument used for measuring the tissue thermal properties relies on the initial verification of the thermal drift of the tissue collecting data for 30 s: if the temperature drift is below a specific threshold (> 0.002 °C/s), the instrument can start the measurement process. The heating needle takes 30 s to warm up (heating time, *t*_h_) and an additional time of 90 s is required by the other needle to measure the temperature variation of the tissue caused by the transfer of heat from the heating needle to the adjacent tissue, so each measurement takes an overall time of 2 min. The obtained data are processed by subtracting the drift rate from the ambient temperature.

The dual needle records the sample's initial temperature (*T*_0_) at the beginning of the heating period; the temperature recorded by the monitoring needle (*T*) is subtracted from *T*_*0*_ to determine the temperature variation needed to solve the Eqs. (3) and (4):

2 $$\Delta T=T-{T}_{0}$$

Knowing the values of physical parameters such as *q*, *r*, which are dependent on the SH-3 dual needle sensor probe characteristics, and *t* and *t*_h_, the *k* (W/m·K) and the *α* (m^2^/s) can be obtained from the Eqs. (3) and (4) using a least squares procedure which minimizes the difference between the model and the measured values:

3 $$ \Delta T = \left[ {\frac{q}{4\pi k}} \right]Ei\left[ { - \frac{r^2}{{4\alpha t}}} \right] \quad t \le t_{{\text{h}}} , $$

4 $$ \Delta T = \left[ {\frac{q}{\pi k}} \right]\left( {E_{i} \left[ { - \frac{{r^{2} }}{{4\alpha \left( {t - t_{{\text{h}}} } \right)}}} \right] - E_{i} \left[ { - \frac{{r^{2} }}{4\alpha t}} \right]} \right)\quad t > t_{{\text{h}}} , $$

where ∆*T* is the temperature rise at the measuring needle (the heating needle increases the tissue temperature by about < 1 °C above the baseline temperature), *q* is defined as the amount of heat released per unit of length (W/m) which derives from the imposition of a certain current at the heating needle for the determined time *t*_h_, i.e., 30 s, in order to locally increase the temperature of the sample, *r* is the distance from the heated needle to the measuring needle which is equal to 6 mm, *t* is time (s). *E*_*i*_(*−x*) is the exponential integral function, which is approximated, in case of small arguments, that is in case of small distances and prolonged observations periods, as the sum of ln*(x)*, i.e., the natural logarithm of the argument, and the Euler–Mascheroni constant, i.e., *γ* ≈ 0.5772.^9,22,39^ The *C*_*v*_ is then calculated with the following formula:

5 $${C}_{v}=\frac{k}{\alpha }$$

Along with the values of the estimated thermal properties, attained through the least squares method, the thermal properties analyzer also provides, at the end of the measurement, a so-called *S*_*yx*_ value. This dimensionless parameter is a measure of the quality of fit as it indicates how well a theoretical heating curve, used as a model, may fit the recorded heating curve. Hence, lower values of *S*_*yx*_ can be used as a metric to assess the goodness of the fit, i.e., a higher similarity of the theoretical and the actual curves, albeit even at higher *S*_*yx*_ results may still be accurate (as specified within the instrument specifications manual). Details on the accuracy of the TEMPOS thermal properties analyzer are provided in the manual of the instrument.^27^

**FIGURE 2:**
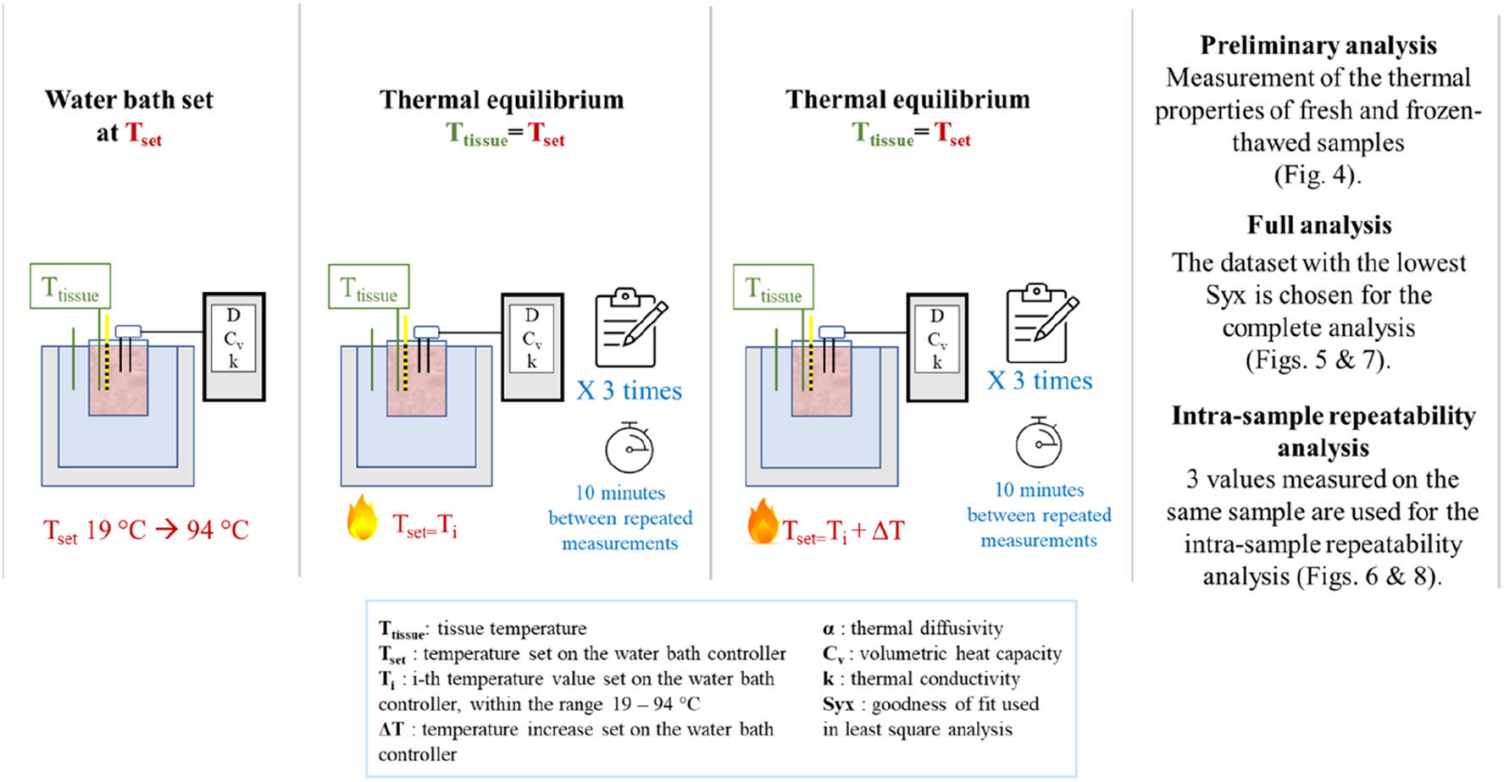
Flowchart of the experiment and the performed analysis.

The measurement protocol was the following: at first, the water bath was set to a series of constant temperatures, in the range from 19 to 94 °C; for each set temperature, three measurements were repeated on the same sample and the same location, to evaluate the measurement repeatability on *α*, *C*_*v*,_ and *k* (intra-sample repeatability analysis, Fig. 2). The consecutive measurements were performed at 10 min time distance, to allow the dual needle to cool down and to avoid that the current, applied to the heating needle, could affect the results. Indeed, after this amount of time, the temperatures recorded by the thermocouples and the other temperature sensors differed by a value < 1 °C from the temperatures recorded prior to performing the measurement, thus showing that the temperature variation due to heating was dissipated. For this procedure, we used three samples for three different temperature ranges for each experimental trial. We performed the measurement acquisition such that the temperature intervals overlapped, and we checked that the last measure of the previous range was in accordance with the first measure of the following temperature range. Moreover, we performed three repetitions of each experimental trial (for a total of nine samples for each tissue type) to also consider the variability among samples, using three different samples for each temperature interval.

Indeed, at the end of the procedure and for each sample, among the three consecutive measures at the same temperature, the one with the smallest *S*_*yx*_ was chosen to perform the full analysis of the temperature dependence of the thermal properties, in the selected measurement range (full analysis in Fig. 2). For each tissue type, this full analysis consisted in the calculation of the average values of thermal properties associated with the lowest *S*_*yx*_ attained in the three experimental trials from room to ablative temperatures to assess the inter samples variability, the estimation of the associated measurement uncertainty, the thermal property modeling through data interpolation and the residuals analysis.

The largest experimental campaign (i.e., full analysis and intra-sample repeatability analysis) was performed on samples that, immediately after the collection of fresh organs (i.e., porcine heart and lung) from a local butcher, were stored at − 20 °C, removed 12 h before the experiment, and placed in a refrigerator at 4 °C to allow the specimen to gradually thaw. The decision to adopt this experimental procedure was due, first, to the fact that an attempt was made to standardize the measurement protocol by keeping the organs always in the same conditions and minimizing the potential effects of deterioration.^3^ The samples were taken on the same day, and a protocol to standardize the storage conditions was implemented. Indeed, given the long times that are required for biological tissue to reach thermal equilibrium at different temperatures and the need to make multiple acquisitions to assess the repeatability of the measurement, there was a risk that the samples could deteriorate/degrade while performing the measurements that covered multiple experimental days for the same organ. Therefore, the technical time required to heat the tissue to thermal equilibrium and measure the thermal properties at different temperatures (i.e., 11 different temperatures for cardiac tissue and 17 temperatures for lung tissue) in the range from room temperature to ablative temperatures, led to favoring the storage protocol used.

For each experimental trial from room to ablative temperatures, we used 3 tissue samples. The experimental trial was repeated 3 times for each tissue type. Overall, we used 5 *ex vivo* hearts for a total of 9 heart tissue samples and 6 *ex vivo* lungs for a total of 9 lung tissue samples for the so-called full analysis and intra-sample repeatability analysis.

Moreover, we have carried out a preliminary analysis by evaluating the thermal properties of fresh *ex vivo* porcine cardiac and lung tissues (i.e., collected from a local butcher, stored at 4 °C and subjected to measurements within 24 h from procurement) at five selected temperatures in the range from room to ablative temperatures. This was performed in order to make a comparison with the thermal properties of frozen-thawed tissues, subjected to the previously described experimental storage procedure, at the same temperatures. For these experimental trials, measurements were repeated three times consecutively (10 min apart between one measurement and the next one) for each temperature value.

### Temperature Measurement Approach

The temperatures of the samples and the thermal bath were measured with two different and complementary approaches.

Four *k*-type thermocouples (0.1 °C accuracy) were used to measure the absolute temperature of the tissue samples and water bath. The first thermocouple was placed in the tissue, at the same depth and close to the dual needle sensor and was used to further verify the temperature of the tissue measured by the SH-3 dual-needle sensor connected to the thermal analyzer (the TEMPOS system is able to resolve temperature to ± 0.001 °C); the second thermocouple was placed on the superficial zone of the sample; the third thermocouple was positioned at around 4 cm depth in the tissue to verify that the temperature of the tissue was homogenous through the whole sample (Fig. 1b); the fourth thermocouple was inserted in the water. In order to assess whether the tissue was in thermal equilibrium, the temperatures registered by the thermocouples and the temperature measured by the dual-needle SH-3 sensor, which is displayed by the thermal analyzer to which it is connected, were compared. Particular attention was paid to ensuring that the temperature registered by the dual-needle SH-3 sensor and the temperature of the thermocouple positioned closer to the dual-needle sensor were in agreement. This was performed since it has been shown in the literature that at least 4 mm of material must be assured to be parallel to the sensor in order to accurately assess the specimen’s thermal properties.^24^ Hence, we wanted to avoid temperature gradients especially in the portion of tissue close to the sensor.

The temperature distribution within the tissue was measured by a 10-sensors metallic needle based on Fiber Bragg Grating (FBG) technology (FiSens GmbH, Braunschweig, Germany).^14,15,34,45^ The 10 sensors have a 1 mm edge-to-edge distance with a sensing length of 1 mm and are embedded in a polyimide-coated fiber, thus covering a length of 19 mm inside the sample.^33,45^ The sensors were interrogated by an industrial optical sensing interrogator (Micron Optics si255 optical interrogator, Micron Optics Inc., Atlanta, USA, 1 pm accuracy corresponding to 0.1 °C) and the optical information was acquired with the ENLIGHT Sensing Analysis Software (Micron Optics Inc., Atlanta, USA). The information provided by the 10 sensors was useful to assess the distribution of the temperature in the tissue and the time required for the sample to reach thermal equilibrium while heated with the thermal bath.

### Measurement Uncertainty

The results for each property and for each set temperature *T*_s_ are expressed in terms of mean value and measurement uncertainty, calculated using the following Eq. (6), and in agreement with the “Guide to the expression of uncertainty in measurement”.^28^

6 $${y}_{Ts}=\overline{{y }_{Ts}}\pm U=\overline{{y }_{Ts}}\pm {k}_{f}\cdot s$$

where ${y}_{Ts}$ indicates the single property, $\overline{{y }_{Ts}}$ is the arithmetic mean, *n* is the number of measurement sets, *U* indicates the uncertainty calculated as the product between the coverage factor *k*_*f*_ and the standard deviation *s*.

The value *k*_*f*_ was obtained from the Student’s t-distribution with a level of confidence of 95%: in our case *n* = 3, since each experimental trial was repeated three times for each tissue type, consequently there are two degrees of freedom, hence the *k*_*f*_ corresponds to a value of 4.30 for both the tissues. The standard deviation *s* is, instead, obtained from the following formula:

7 $$s=\sqrt{\frac{{{\sum }_{i=1}^{n}\left({y}_{Ts,i}-\overline{{y }_{Ts}}\right)}^{2}}{n\left(n-1\right)}}$$

### Thermal Property Modeling

The behavior of the measured *α* and *k* of the heart and lung as well as *C*_*v*_ for heart tissue can be described by exponential curves^25^ through the analysis and the modeling of the obtained data by the following equation:

8 $$y\left(T\right)=a+b\cdot \mathrm{exp}\left(cT\right)$$

while the following linear equation was used for describing the trend of lung tissue *C*_*v*_:

9 $$y\left(T\right)=m\cdot T+q$$

These curves of best fit essentially represent fitting models where *y(T)* represents the temperature-dependent thermal property, i.e., *k*, *α*, or *C*_*V*_, and *T* is the temperature recorded by SH-3 dual-needle sensor connected to the thermal analyzer. The values *a, b, c,* and *m, q* are the coefficients of the equation in the best data fitting and were derived using MATLAB® software (MathWorks, Natick, MA, USA) employing the least squares method.^39^ The coefficient, R^2^, and the analysis of the residuals were used to evaluate the goodness of the fitting and the chosen models. In the analysis of the residuals, the residuals were calculated as the difference between the measured property and the value calculated with (8) or (9), at each temperature value.

The intra-sample repeatability analysis has been performed for each sample and each set temperature. A total of 3 consecutive measurements of the thermal properties were performed, with a time-lapse of 10 min between each acquisition. The mean value and the standard deviation of the three repetitions were calculated to assess the repeatability of the measurement.

## Results

### Temperature Distribution in the Samples

**FIGURE 3:**
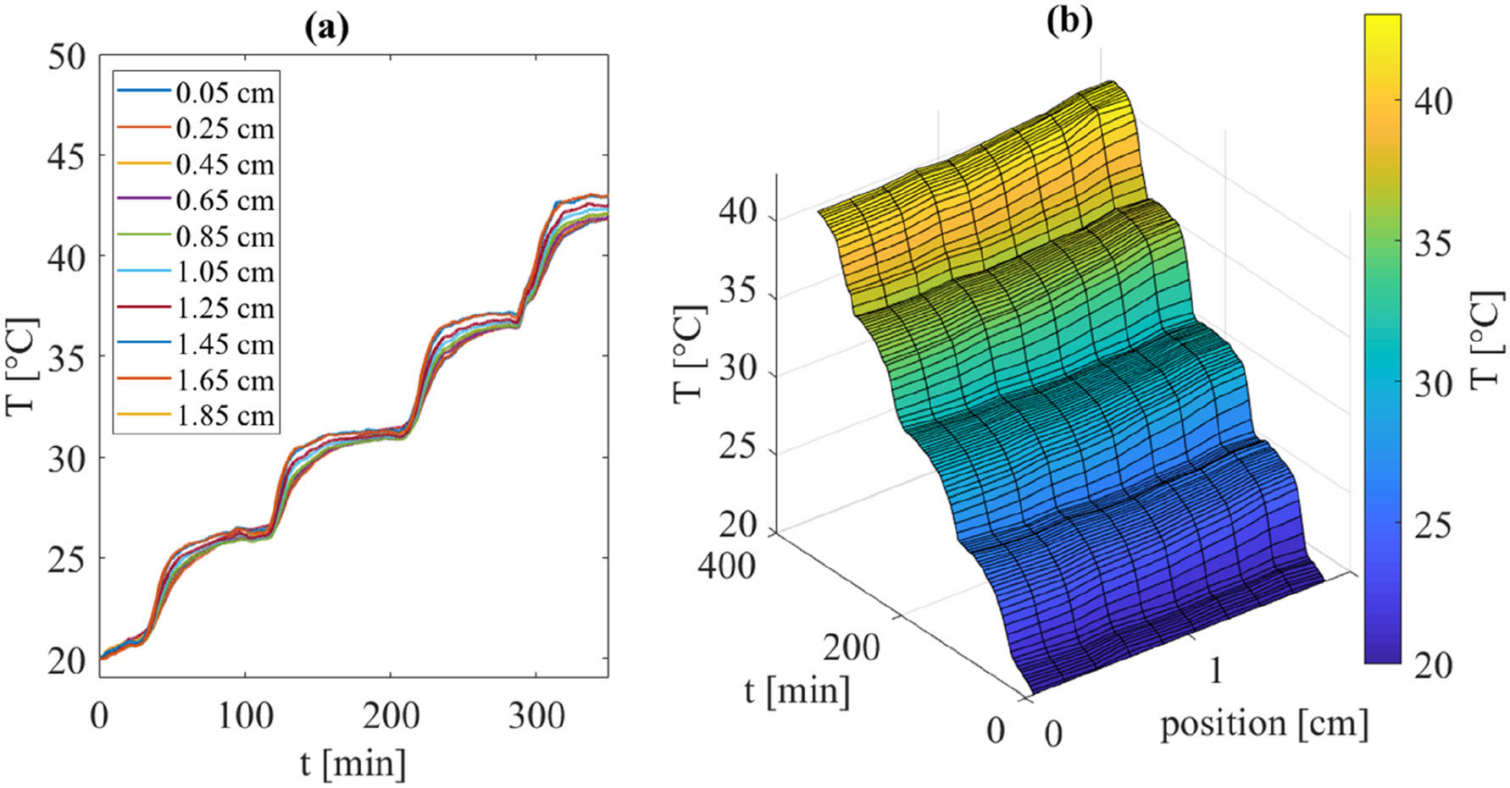
Temperature distribution as a function of time in the samples across the tissue depths: (a) temperature trends measured by the 10 FBG sensors and (b) temperature map across the sensors and in time. As an example, the results of one of the tests on the heart are reported.

The temperature distribution measured with FBGs during single experiments and at different sample depths indicate that the tissue reached equilibrium after about 50 min (Fig. 3). For this reason, the tissue was maintained about 80 min before performing the first measurement of the thermal properties. Other 20 min were kept allowing to carry out the second and third readings, and then the temperature of the water bath was increased to the next set temperature value.

### Preliminary Analysis on Fresh and Frozen-Thawed Tissues

**FIGURE 4:**
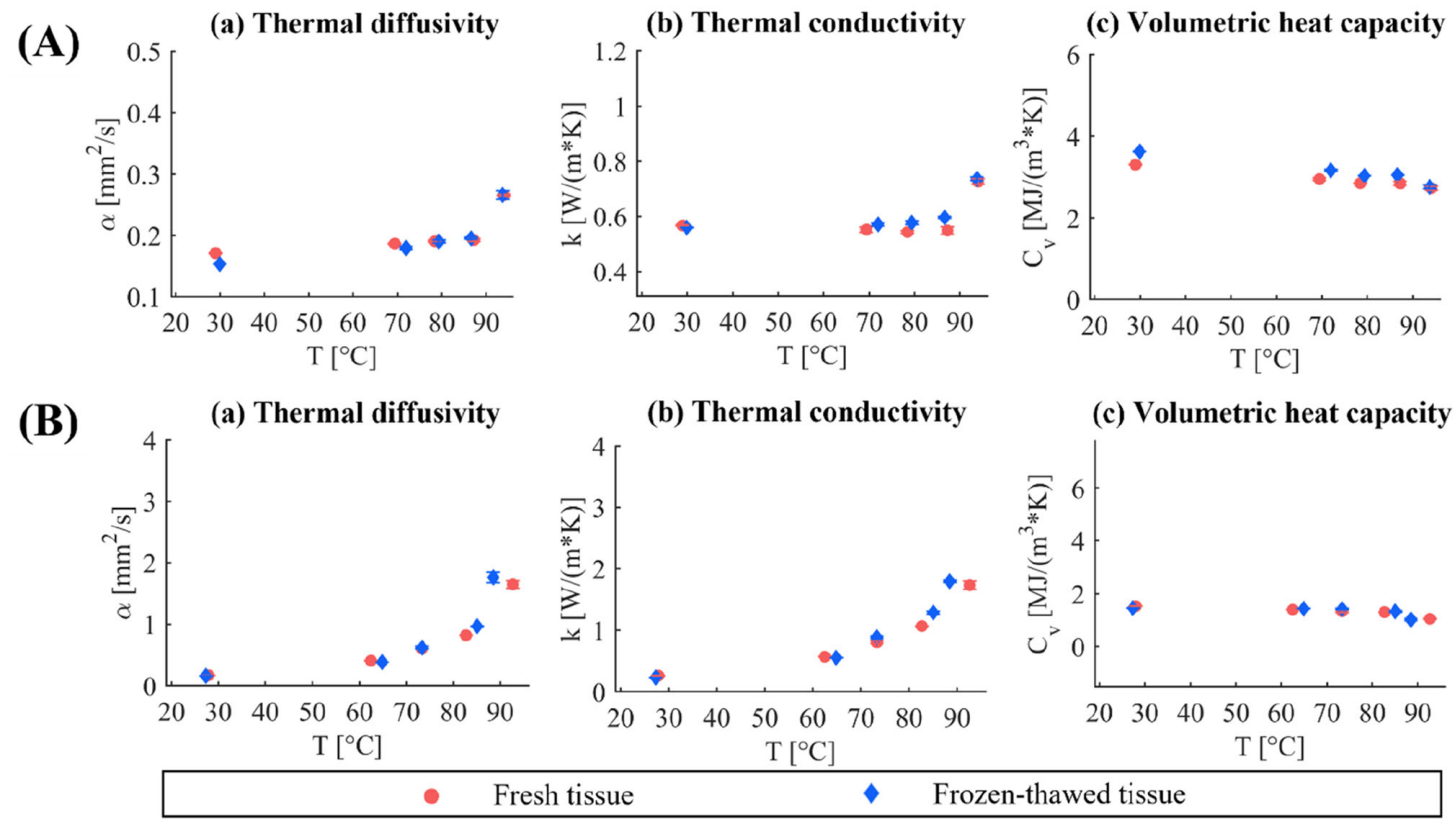
Preliminary analysis on fresh and frozen-thawed tissues. (A) thermal properties of heart tissue: (a) thermal diffusivity, (b) thermal conductivity, and (c) volumetric heat capacity. (B) thermal properties of lung tissue: (a) thermal diffusivity, (b) thermal conductivity, and (c) volumetric heat capacity. The results of the mean values and the standard deviation of the three consecutive repetitions, at each temperature, are shown from room to ablative temperatures.

The results of the preliminary analysis conducted to investigate the thermal properties of fresh and frozen-thawed tissue samples, from room up to ablative temperatures, are depicted in Fig. 4. Figure 4(A) shows the average values of *α*, *k,* and *C*_*v*_ and the associated standard deviations for the three consecutive measurement repetitions, at each temperature, for heart tissue. Besides, Fig. 4(B) reports the mean values of the thermal properties and standard deviations attained in the case of deflated lung tissues. For both the cardiac and pulmonary tissues, no substantial differences emerged between the thermal properties of fresh and frozen-thawed samples, showing similar values not only at nominal conditions (room temperature) but also at elevated temperatures. For instance, the maximum percentage difference between the thermal properties of fresh and frozen-thawed heart tissues was < 2% at 94 °C. Similarly, for lung tissue, the maximum percentage difference of the average values of the thermal properties measured for fresh specimens and samples undergoing the freezing–thawing procedure was < 7%, at ablative temperatures close to 90 °C.

### Thermal Properties of the Heart

**FIGURE 5:**
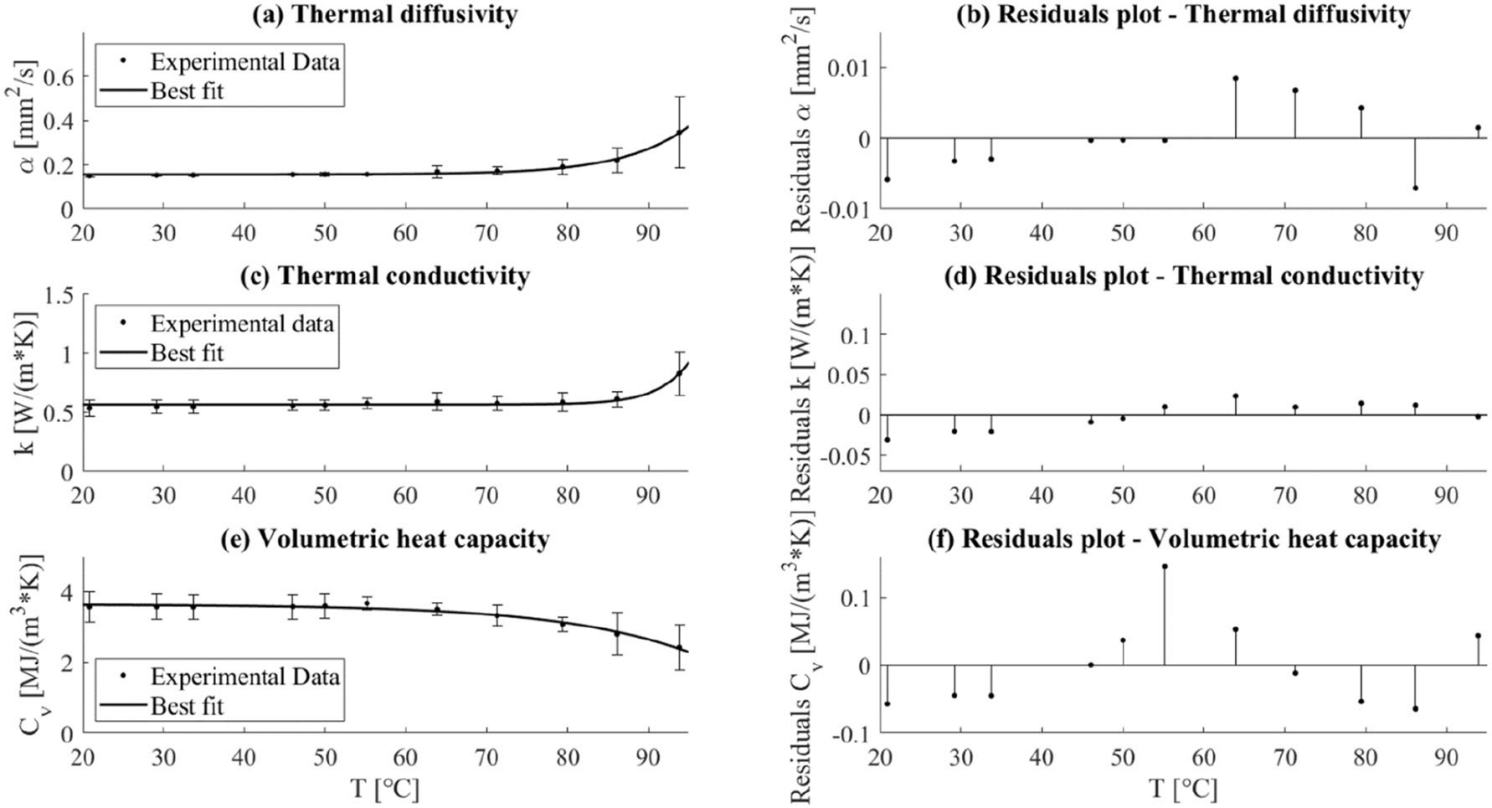
Thermal properties for *ex vivo* porcine heart as a function of temperature: the average values of the thermal properties, the associated measurement uncertainty, and the best fitting curves for (a) thermal diffusivity, (c) thermal conductivity, and (e) volumetric heat capacity are displayed as well as the plots derived from the analysis of the residuals (b, d, f).

The thermal properties of the *ex vivo* porcine heart tissue were measured over a temperature range of 21–94 °C. Figure 5 shows the average values of each property under analysis and the associated measurement uncertainty with a 95% confidence level, for the different temperatures. The *x*-axis against which the variance of the thermal properties is estimated is defined by the temperature measured by the SH-3 dual-needle sensor connected to the thermal analyzer.

The *α* associated with the heart tissue showed an approximately constant behavior up to ~ 70 °C followed by a gradual increase until about 80 °C and a more substantial increase up to 94 °C. Increments of 27% and 131% with respect to the initial value (i.e., 0.150 mm^2^/s) were assessed at temperatures of 79 °C and 94 °C, respectively. Besides, the observed trend for *k* was almost constant until around 80 °C, then *k* slightly increased (i.e., 0.612 W/(m·K) at 86 °C), and it reached its maximum at 94 °C with a value of 0.828 W/(m·K), corresponding to an increase of 55% from the nominal value at 21 °C. Regarding *C*_*v*_, its behavior was rather constant until 55 °C, afterwards, *C*_*v*_ subsided until 94 °C, showing, at the latter temperature, a decrease of 32% compared to its baseline value at 21 °C.

To describe the variation of the thermal properties of the heart as a function of temperature, the mathematical curves that most accurately approximate (best fit) the trend of the experimentally measured properties over temperature were derived using equation (8). Regression coefficients, R^2^, and coefficients of equation (8) used to best fit the trend of the thermal properties of the heart over temperature are reported in Table 1.

**TABLE 1:** Regression coefficients, R^2^ and coefficients of Eq. (8).

Thermal property	*a*	*b*	*c*	${R}^{2}$
Thermal diffusivity *α* $\left(\frac{{\mathrm{mm}}^{2}}{s}\right)$	0.1555	1.432 × 10^−6^	0.1256	0.9928
Thermal conductivity $k \left(\frac{W}{m\cdot K}\right)$	0.5655	5.034 × 10^−12^	0.263	0.9551
Volumetric heat capacity ${C}_{v} \left(\frac{MJ}{{m}^{3}\cdot K}\right)$	3.643	− 0.003533	0.06263	0.9743

As far as concerns the residuals analysis, the residuals are distributed around the y = 0 value, and the average mean value of the residuals is < 5% of the measured property value. Especially for *k* and *C*_*v*_, the curves (8) slightly overestimate the values in the range 21—34 °C, then an underestimation occurs from 50 to 64 °C, and lastly, an overestimation is again appreciable, with the exception of the last value measured at 94 °C.

**FIGURE 6:**
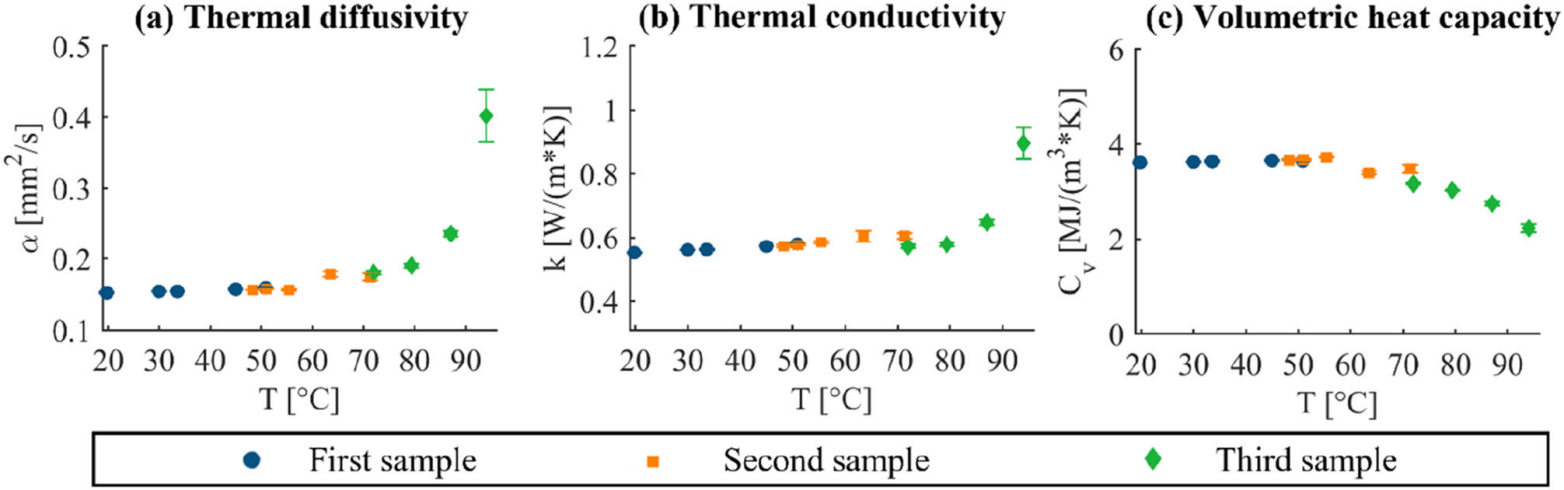
Intra-sample repeatability analysis for the heart tissue: (a) thermal diffusivity, (b) thermal conductivity, and (c) volumetric heat capacity. The results of the mean values and the standard deviation of the three consecutive repetitions are representatively shown for three heart tissue samples of one experimental trial from room to ablative temperatures (first sample: from room temperature to ~ 51 °C, second sample: from 48 to ~ 71 °C, third sample: from 71 to 94 °C).

The intra-sample repeatability analysis shows good repeatability of the measurement, denoted by the low standard deviation attained for the three measurement repetitions at each temperature. A slighter increase in the standard deviation of the mean is registered for the *α* and *k* of the heart tissue at 94 °C (Fig. 6).

### Thermal Properties of the Lung

**FIGURE 7:**
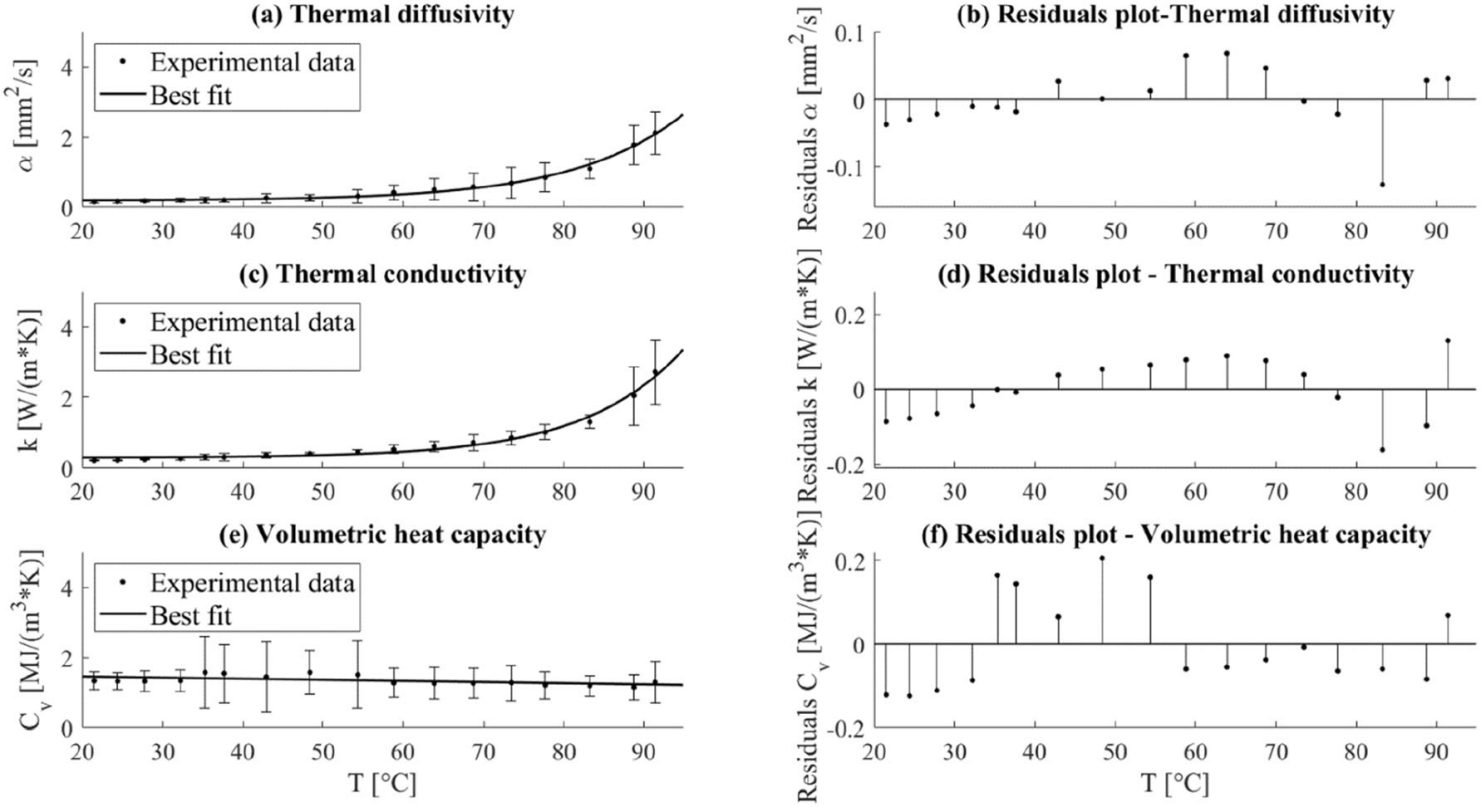
Thermal properties, i.e., (a) thermal diffusivity, (b) thermal conductivity, and (c) volumetric heat capacity of *ex vivo* porcine lung tissue as a function of temperature and their associated measurement uncertainty. The best-fitting curves interpolating the experimental data are depicted (a, c, e) as well as the plots of the residuals over temperature (b, d, f).

In the case of the thermal properties of porcine lung tissue, the measurements were performed at temperatures ranging between 22 and 91 °C. The results regarding the evaluation of the temperature dependence of the thermal properties of lung tissue are depicted in Fig. 7, along with the analysis of the residuals. The horizontal axis against which the variance of the thermal properties of lung tissue is estimated is defined by the temperature measured by the SH-3 dual-needle sensor.

As it is possible to observe, the *α* associated with the *ex vivo* lung tissue exhibited an exponential rise with temperature, denoting a marked increment at ablative temperatures, *e.g.*, at temperatures of 83, 89, and 91 °C, *α* respectively increased by 7.1, 11.5, and 13.7 times compared to its baseline value at room temperature (i.e., 0.155 mm^2^/s). Likewise, the value of *k* was subject to an exponential increase with temperature. From the nominal value of 0.207 W/(m·K) measured at 22 °C, *k* rose by 6.3, 9.9, and 13.1 times, at 83, 89, and 91 °C, respectively; thus, reaching a maximum value of 2.721 W/(m·K).

Differently from the other two thermal properties, *C*_*v*_ showed no substantial fluctuations within the investigated temperature range, registering values of 1.33 MJ/(m^3^·K) and 1.30 MJ/(m^3^·K) at room temperature and at 91 °C, accordingly.

In the analysis of the R^2^ parameter, *α* and *k* show a value close to 1, so the exponential model can be considered an accurate model for the approximation of the experimental data attained for these thermal properties. In the case of *C*_*V*_, the curve which most accurately approximates the experimental measurements at various temperatures is given by the linear equation (9) (Table 2).

**TABLE 2:** Regression coefficients, R^2^ and coefficients of Eqs. (8) and (9).

Thermal property	*a*	*b*	*c*	${R}^{2}$
Thermal diffusivity *α* $\left(\frac{{\mathrm{mm}}^{2}}{s}\right)$	0.1815	0.002224	0.0739	0.9938
Thermal conductivity $k \left(\frac{W}{m\cdot K}\right)$	0.2852	0.001288	0.08196	0.987
Thermal property	*m*	*q*		
Volumetric heat capacity ${C}_{v} \left(\frac{MJ}{{m}^{3}\cdot K}\right)$	− 0.003253	1.526		

Equation (8) was utilized to mathematically fit the best curves to represent the temperature dependence of the thermal diffusivity and thermal conductivity associated with lung tissue, whereas Eq. (9) was used to approximate the profile of the volumetric heat capacity over temperature

Also for the lung tissue, the residuals are distributed around the *y* = 0 value, and the average mean value of the residuals is < 10% of the property value. For instance, for *k* and *α*, the curve (8) slightly overestimates the values in the range 22–38 °C, then an underestimation occurs up to 74 °C, and lastly, an overestimation is again appreciable, except for the last values measured at a temperature > 90 °C.

**FIGURE 8:**
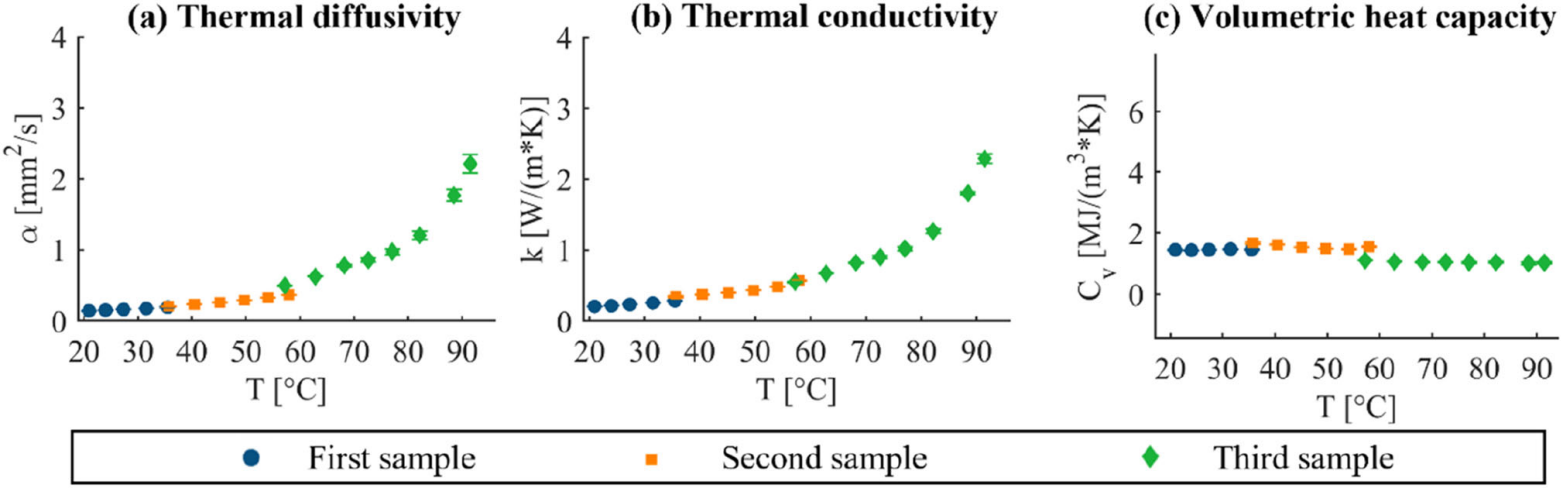
Analysis of the intra-sample measurement repeatability for the *ex vivo* lung tissue. For (a) thermal diffusivity, (b) thermal conductivity, and (c) volumetric heat capacity, the mean values and the standard deviation of the three consecutive measurement repetitions are representatively shown for three lung tissue samples of one experimental trial from room to ablative temperatures (first sample: from room temperature to ~ 36 °C, second sample: from 36 to ~ 58 °C, third sample: from 57 to 92 °C).

Regarding the evaluation of the intra-sample measurement repeatability, the observed low standard deviation values associated with the three repetitions performed at the same temperature on the same tissue specimen suggest good repeatability of the measure (Fig. 8).

## Discussion

The purpose of this study was to characterize the thermal properties of heart and lung tissues, considering their temperature dependence. Thus, we exploited a dual-needle technique based on the transient hot-wire method^24^ to perform the first-ever detailed estimation of the thermal properties of *ex vivo* porcine cardiac and pulmonary tissues in the hyperthermia and ablative temperature ranges. The dual-needle sensor-based technique has been already utilized by our group^11,43^ and other researchers^25,39,49,50^ for the measurement of the thermal properties of biological tissues subjected to temperature variation. In this work, by employing an ASTM 5334- e IEEE 442-compliant thermal properties analyzer, manufactured according to the ISO 2008 standards,^27^ we were able to characterize the selected tissues in terms of *α*, *k* and *C*_*v*_.

We first performed a preliminary analysis on the thermal properties of fresh and frozen-thawed tissue, at different selected temperatures, which highlighted no substantial differences between the tissues undergoing these different conservation protocols. The attained results are in accordance with previous experiments on different biological media (i.e., pancreas tissues) which showed, differently from other physical properties such as optical properties^3,35^, no significant variation in terms of thermal conductivity for fresh and frozen (for 7 and for 14 days) specimens.^3^ These results may be useful for the improvement of laboratory protocols regarding tissue conservation for better accountability of experimental measurements. Then, the full analysis to evaluate inter samples variability and the intra-sample repeatability analysis were performed.

Concerning the thermal properties of heart tissue, we measured *α*, *k,* and *C*_*v*_ in a temperature interval between 21 and 94 °C, thus expanding the temperature range considered in previous studies.^10,55^ We first validated our measurement approach by comparing the results attained at ambient temperature with the values reported in previous works which employed self-heated thermistor probes for the thermal properties characterization (Table 3).^10,55^ The average values of *α*, *k* and *C*_*v*_ obtained at a mean temperature of 21 °C were 0.150 mm^2^/s, 0.535 W/(m·K), 3.57 MJ/(m^3^·K), respectively, which are in accordance with the values reported by Bhavaraju and Valvano for porcine myocardium at 25 °C (i.e., 0.152 mm^2^/s, 0.515 W/(m·K), and 3.39 MJ/(m^3^·K))^10^ and by Valvano *et al.* at 23 °C for porcine myocardium (i.e., 0.139 mm^2^/s, 0.515 W/(m·K), and 3.71 MJ/(m^3^·K)) and human myocardium (i.e., 0.140 mm^2^/s, 0.520 W/(m·K), and 3.70 MJ/(m^3^·K)).^55^ Subsequently, we assessed the temperature dependence of the heart tissue thermal properties in the hyperthermic and ablative temperature range. We observed that the values of *α* remained almost constant until around 70 °C and gradually changed until ~ 80 °C. Above ~ 80 °C, a more substantial variation in this thermal property was registered. Similarly, the *k* remained almost constant up to ~ 80 °C, then, above this temperature, it gradually increased up to 86 °C and was subjected to a steeper rise at 94 °C. The attained results are in line with the observations reported by Bhavaraju and Valvano, who investigated the *α*, *k*, and density in *ex vivo* porcine myocardial tissue at 25, 37, 50, 62, and 76 °C.^10^ In this temperature interval, they observed a decrease of *k* as a function of temperature, whose average values were 0.515 W/(m·K) at 25 °C and 0.476 W/(m·K) at 76 °C. However, the variations of *k* and *α* were not significant (according to a variance of the data at a 5% level of significance), in the mentioned range, despite a significant loss in the water content due to temperature increase over 50 °C (Table 3).

**TABLE 3:** Thermal properties of the heart as a function of temperature attained in the present work and in other literature studies (for the studies of *Bhavaraju and Valvano, and Valvano et al.* the values of *C*_*v*_ were calculated by *C*_*v*_ = k/α as they were not directly reported in their studies).

Temperature [°C]	Result of this work (*ex vivo* porcine heart)	Bhavaraju and Valvano (*ex vivo* porcine myocardium)^10^	Valvano *et al. *(*ex vivo* porcine myocardium)^55^	Valvano *et al. *(*ex vivo* human myocardium)^55^
Thermal diffusivity (mm^2^/s)				
3	–	–	0.129	0.130
10	–	–	0.133	0.339
17	–	–	0.136	0.137
21	0.150	–	–	–
23			0.139	0.140
25	–	0.152	–	–
29	0.152	–	–	–
30			0.142	0.144
34	0.153	–	–	–
37	–	0.161	0.146	0.147
45			0.150	0.151
46	0.156	–	–	–
50	0.156	0.171	–	–
55	0.157	–	–	–
62	–	0.171	–	–
64	0.168	–	–	–
71	0.173	–	–	–
76	–	0.179	–	–
79	0.191	–	–	–
86	0.220	–	–	–
94	0.346	–	–	–
Thermal conductivity (W/(m·K))				
3	–	–	0.488	0.496
10	–	–	0.497	0.505
17	–	–	0.507	0.513
21	0.535	–	–	–
23	–	–	0.515	0.520
25	–	0.515	–	–
29	0.545	–	–	–
30	–	–	0.524	0.528
34	0.545	–	–	–
37	–	0.531	0.533	0.537
45	–	–	0.544	0.546
46	0.557	–	–	–
50	0.561	0.510	–	–
55	0.576	–	–	–
62	–	0.493	–	–
64	0.589	–	–	–
71	0.576	–	–	–
76	–	0.476	–	–
79	0.586	–	–	–
86	0.612	–	–	–
94	0.828	–	–	–
Volumetric heat capacity (MJ/(m^3^·K))				
3	–	–	3.798	3.804
10	–	–	3.766	3.767
17	–	–	3.735	3.732
21	3.572	–	–	–
23	–	–	3.711	3.704
25	–	3.388	–	–
29	3.576	–	–	–
30	–	–	3.683	3.672
34	3.568	–	–	–
37	–	3.298	3.657	3.641
46	3.580	–	3.628	3.608
50	3.598	2.983	–	–
55	3.676	–	–	–
62	–	2.883	–	–
64	3.502	–	–	–
71	3.324	–	–	–
76	–	2.659	–	–
79	3.078	–	–	–
86	2.800	–	–	–
94	2.424	–	–	–

It is worth noticing that according to our results, the cardiac tissue *α* and *k* remain constant at temperatures relevant from a biological point of view such as temperatures within the hyperthermia range and also at temperatures close to the threshold for the instantaneous thermal damage in tissue (i.e., 60 °C).^20^ However, at higher temperatures (> 90 °C), which can be easily reached close to the energy-delivery applicator during thermal ablation procedures, the tissue *α* and *k* experience a steep rise which may in turn affect the heat distribution within the biological tissue during treatment. Differently from the profiles of *α* and *k*, *C*_*v*_ was almost constant until 55 °C and gradually decreased until ~ 80 °C; above 80 °C the decrease became more marked.

Regarding the thermal properties of *ex vivo* pulmonary tissue, we investigated their temperature dependence up to 91 °C, hence providing a first comprehensive analysis of the thermal dependence of *α*, *k* and *C*_*v*_ associated with lung tissue, up to ablative temperatures. The results attained at room temperature (i.e., 22 °C) were 0.155 mm^2^/s, 0.207 W/(m·K), 1.33 MJ/(m^3^·K), for *α*, *k,* and *C*_*v*_, accordingly. These measurements are in line with the values registered by Silva *et al.* on *ex vivo* lung samples at 22.11 °C (i.e., *α* = 0.14 mm^2^/s, *k* = 0.26 W/(m·K), *C*_*v*_ = 1.84 MJ/(m^3^·K)) (Table 4).^48^ In the mentioned study, Silva *et al.* estimated the thermal properties of ovine lung, by means of the dual needle technique, at room and body temperatures. They observed that the lung presents similar *α*, but lower *k* and *C*_*v*_ in comparison to other tissues.^48^ Our results are in accordance with these observations. Indeed, at room temperature, the heart tissue presented thermal properties which are comparable to other tissues, such as liver,^47,48^ and other muscle (i.e., superior leg muscle) tissues.^48^ Conversely, the *k* and *C*_*v*_ associated with lung tissue were substantially lower (*e.g.*, ~ 21–22 °C, the *k* of the heart was 2.6-fold higher compared to the value of the lung tissue, whereas the *C*_*v*_ associated with the heart was 2.7-fold higher compared to the same property for the lung).

**TABLE 4:** Thermal properties of lung tissue as a function of temperature obtained in the present work and in other literature studies (for the studies of *Valvano et al.*, the values of *C*_*v*_ were calculated by *C*_*v*_ = k/α as they were not directly reported in their study).

Temperature [°C]	Result of this work (*ex vivo* porcine lung)	Valvano *et al. *(*ex vivo* porcine lung)^55^	Valvano *et al. *(ex vivo human lung)^55^	Silva *et al. *(*ex vivo* ovine lung)^48^
Thermal diffusivity (mm^2^/s)				
3	–	0.072	0.120	–
10	–	0.078	0.122	–
17	–	0.083	0.125	–
22	0.155	–	–	0.14
23	–	0.088	0.126	–
24	0.165	–	–	–
26	–	–	–	0.15
28	0.177	–	–	–
30	–	0.094	0.129	
32	0.196	–	–	–
35	0.200	–	–	–
37	–	0.099	0.131	–
38	0.199	–	–	0.16
43	0.261	–	–	–
45	–	0.106	0.133	–
48	0.262	–	–	–
50	–	–	–	–
54	0.318	–	–	–
59	0.418	–	–	–
64	0.499	–	–	–
69	0.585	–	–	–
73	0.686	–	–	–
78	0.853	–	–	–
83	1.101	–	–	–
89	1.780	–	–	–
91	2.120	–	–	–
Thermal conductivity (W/(m·K))				
3	–	0.241	0.411	–
10	–	0.256	0.419	–
17	–	0.272	0.427	–
22	0.207	–	–	0.26
23		0.285	0.434	–
24	0.217	–	–	–
26	–	–	–	0.28
28	0.233	–	–	–
30	–	0.300	0.442	
32	0.259	–	–	–
35	0.307	–	–	–
37	–	0.316	0.451	–
38	0.305	–	–	0.32
43	0.366	–	–	–
45	–	0.334	0.460	
48	0.406	–	–	–
50	–	–	–	–
54	0.460	–	–	–
59	0.523	–	–	–
64	0.615	–	–	–
69	0.721	–	–	–
73	0.854	–	–	–
78	1.013	–	–	–
83	1.308	–	–	–
89	2.047	–	–	–
91	2.721	–	–	–
Volumetric heat capacity (MJ/(m^3^·K))				
3	–	3.346	3.418	–
10	–	3.304	3.425	–
17	–	3.268	3.431	–
22	1.334	–	–	1.84
23	–	3.241	3.437	–
24	1.322	–	–	
26	–	–	–	1.94
28	1.324	–	–	–
30	–	3.213	3.443	–
32	1.334	–	–	–
35	1.576	–	–	–
37	–	3.188	3.449	–
38	1.548	–	–	2.14
43	1.451	–	–	–
45	–	3.162	3.455	–
48	1.574	–	–	–
50	–	–	–	–
54	1.509	–	–	–
59	1.275	–	–	–
64	1.263	–	–	–
69	1.264	–	–	–
73	1.280	–	–	–
78	1.208	–	–	–
83	1.195	–	–	–
89	1.153	–	–	–
91	1.297	–	–	–

Both the *α* and *k* of lung tissue were characterized by an exponential increase with temperature. Conversely, no substantial variations were observed for the *C*_*v*_, whose minimum and maximum values were 1.20 MJ/(m^3^·K) and 1.58 MJ/(m^3^·K), correspondingly. It is worth mentioning that, differently from what was observed for cardiac tissue, already at a temperature close to those that mark instantaneous thermal damage in tissue (i.e., 64 °C), *α* and *k* of lung tissue were found to vary about 3 times from the nominal value at room temperature. The marked change in *α* and *k* due to temperature elevation is emphasized by the fact that, at temperatures > 90 °C, *α* and *k* associated with lung are greater than those associated with cardiac tissue, showing respectively 6.1-fold and 3.3-fold higher values compared to heart tissue at 94 °C. The observed increase in *α* and *k* with temperature for lung tissue was higher compared to also other organs such as liver, kidney, and brain, which were subject of previous studies.^12,43,49,50^ For both the heart and lung tissues, the measurement uncertainty related to *α* and *k* was found to increase at higher temperatures, which is also in accordance with previous experimental investigations.^43,49^

The mechanisms underlying the temperature-dependent variation of thermal properties in biological media are yet to be fully enlightened, however, the observed change in thermal properties in heart and lung tissue may be ascribable to the cellular and subcellular modifications in tissue due to temperature change. Indeed, the temperature increment involves the escape of the extracellular water content from tissue, the modification of the protein structures due to the breaking of hydrogen bonds, and the alteration of cell membranes allowing water to leak out into the extra-cellular space.^10^ Approaching the water phase transition temperature, the water evaporation phenomenon initiates, together with vapor diffusion and condensation in other districts, characterized by lower local pressure, of the same tissue.^21,39^ These phenomena may alter the overall tissue capability to transfer and retain heat compared to nominal conditions. As observed in other studies for liver tissue,^39^ also in our investigation, the most prominent variations in *α* and *k* were attained at temperatures > 91 °C. The marked variation of the *α* and *k* for the lung tissue with temperature, in comparison to the heart, and other tissues,^12,47^ may be due to the peculiar porous structure and characteristics of the lung.^41^ As a matter of fact, at baseline conditions, studies have shown a similar water content for lung tissue compared to other organs, while its density is around half of the value determined for the other tissues (*e.g.*, liver, kidney, and muscle tissue).^48^

In conclusion, in this work, we devised the analysis of the temperature dependence of thermal properties of heart and lung tissues. The best-fit regression curves based on the experimentally measured data are proposed to be utilized in predictive mathematical frameworks toward optimized preplanning of hyperthermic and ablative thermal procedures involving heart and lung tissues. Indeed, the inclusion of temperature-dependent properties in computational models of the bioheat transfer may help enhance the accuracy of the prediction in terms of estimation of temperature distribution and final tissue thermally coagulated zone.^40^ An accurate estimation of the delivered thermal dose could in turn be beneficial for the minimization of procedural downsides, such as steam pops and hematic clots formation during catheter-mediated ablation for the treatment of cardiac arrhythmias,^56^ and the precise thermal eradication of lung neoplasms without excessive damage to the surrounding healthy tissue.^7^ Furthermore, the reported thermal properties could be useful for the refinement and validation of medical devices that must interface with heart and lung tissue, and for the realization of phantoms mimicking the behavior of these tissues.

Future studies should be envisaged to further enlighten the mechanisms of heat transfer in tissue and the temperature-induced variation of the thermal properties in the biological media in the hyperthermia and ablative temperature intervals. Future works should also consider including in the experimental procedure more physiological-like conditions. An example is given by the air normally present in the *in vivo* lungs, as the thermal properties of inflated lungs might be decreased by the presence of the air,^26,58^ thus affecting the heat transfer throughout the tissue. Moreover, although porcine tissue exhibits high morphological and histological similarity with human tissue,^31^ the evaluation of the temperature sensitivity of tissue thermal properties should be also expanded to human healthy and tumorous tissues. Finally, the temperature dependence of thermal properties should be assessed during *in vivo* trials, to include the effect of blood perfusion and other metabolism-related contributions on the recovered measurements.
