# Correction: Temperature Dependence of Thermal Properties of *Ex Vivo* Porcine Heart and Lung in Hyperthermia and Ablative Temperature Ranges

**DOI:** 10.1007/s10439-023-03161-w

**Published:** 2023-02-18

**Authors:** Leonardo Bianchi, Martina Bontempi, Sabrina De Simone, Martina Franceschet, Paola Saccomandi

**Affiliations:** Department of Mechanical Engineering, Politecnico di Milano, 20156 Milan, Italy

**Correction: Annals of Biomedical Engineering** 10.1007/s10439-022-03122-9

In the original article, the paragraphs between Eqs. (6) and (7) erroneously refer to s as the standard deviation. The corrected paragraphs should read as follows:

6 $${y}_{Ts}=\overline{{y }_{Ts}}\pm U=\overline{{y }_{Ts}}\pm {k}_{f}\cdot s$$

where *y*_*Ts*_ indicates the single property, $\overline{{y }_{Ts}}$ is the arithmetic mean, U indicates the uncertainty calculated as the product between the coverage factor *k*_*f*_ and *s*.

The value *k*_*f*_ was obtained from the Student’s t-distribution with a level of confidence of 95%: in our case, the number of measurements is n = 3, since each experimental trial was repeated three times for each tissue type, consequently there are two degrees of freedom, hence the *kf* corresponds to a value of 4.30 for both the tissues. Here, *s* is obtained from the following formula:

7 $$s=\sqrt{\frac{{{\sum }_{i=1}^{n}\left({y}_{Ts,i}-\overline{{y }_{Ts}}\right)}^{2}}{n\left(n-1\right)}}$$

